# Assortative mating and gene flow generate clinal phenological variation in trees

**DOI:** 10.1186/1471-2148-12-79

**Published:** 2012-06-08

**Authors:** Jean-Paul Soularue, Antoine Kremer

**Affiliations:** 1 INRA, UMR 1202 BIOGECO, Cestas F-33610, France; 2 Univ. Bordeaux, BIOGECO, UMR 1202, Talence F-33400, France

## Abstract

**Background:**

On-going climate change is shifting the timing of bud burst (TBB) of broad leaf and conifer trees in temperate areas, raising concerns about the abilities of natural populations to respond to these shifts. The level of expected evolutionary change depends on the level and distribution of genetic variation of TBB. While numerous experimental studies have highlighted the role of divergent selection in promoting clinal TBB differentiation, we explored whether the observed patterns of variation could be generated by the joint effects of assortative mating for TBB and gene flow among natural populations. We tested this hypothesis using an *in silico* approach based on quantitative genetic models.

**Results:**

Our simulations showed that genetic clines can develop even without divergent selection. Assortative mating in association with environmental gradients substantially shifted the mean genetic values of populations. Owing to assortative mating, immigrant alleles were screened for proximal or distant populations depending on the strength of the environmental cline. Furthermore, we confirmed that assortative mating increases the additive genetic variance within populations. However, we observed also a rapid decline of the additive genetic variance caused by restricted gene flow between neighboring populations resulting from preferential matings between phenologically-matching phenotypes.

**Conclusions:**

We provided evidence that the patterns of genetic variation of phenological traits observed in forest trees can be generated solely by the effects of assortative mating and gene flow. We anticipate that predicted temperature increases due to climate change will further enhance genetic differentiation across the landscape. These trends are likely to be reinforced or counteracted by natural selection if phenological traits are correlated to fitness.

## Background

Apical bud phenology of temperate trees has been intensively studied in recent years owing to predicted shifts in the timing of bud development as a result of climate changes [1]. Monitoring of leaf unfolding in various species across their distributions has shown that global warming will trigger earlier flushing [2-4]. These observations have raised concerns about the capacity of tree populations to cope with changes in the timing of bud burst (TBB), which is related to the fitness of trees in two ways: *(i)* it establishes the length of the growing season and is a major determinant of growth [5], *(ii)* it determines the timing of flowering, so is related to fecundity [6]. The adaptive response of TBB to global warming is dependent on the level and distribution of genetic variation within a species; the more variation, the larger the predicted genetic shift in TBB. Numerous investigations involving common garden experiments have demonstrated that TBB exhibits large intra- and inter-population differences, as shown by high population differentiation (*Q*_*ST*_) associated with high heritability values [7]. Additional genetic investigations indicated that juvenile-mature correlation in TBB is high and genotype-environment interactions are low [8]. Finally, genetic dissection by quantitative trait loci (QTLs) mapping has shown that many QTLs contribute to TBB, but these QTLs show stable expression over years and sites [9].

Regardless of species, TBB follows strong geographic clinal patterns of variation, either altitudinal, latitudinal or longitudinal. Phenotypic clines revealed by *in situ* observations of TBB show congruent patterns across species: bud burst in southern latitudes or lower altitudes occurs earlier than in northern latitudes or higher altitudes [10-12], because TBB is triggered by heat sum [13]. Genetic clines can be assessed in common garden experiments where TBB is observed under the same environmental conditions for all populations and are illustrated by the linear relationships between TBB of different populations and geographic variables. Interestingly, genetic clines vary across species and exhibit co-gradient variation or counter-gradient variation with geographic variables and associated phenotypic clines [14,15]. Co-gradient variation corresponds to clines of both phenotypic variation and genetic variation in a species that co-vary in the same way with the environmental gradient. Counter-gradient variation occurs when phenotypic and genetic clines vary in opposite directions. In the case of oak, genetic and phenotypic clines exhibit co-gradient variation; *e.g.* populations from southern latitudes flush earlier than populations from northern latitudes, when assessed under the same conditions in common gardens [16,17]. In the case of beech, genetic clines are opposite to phenotypic clines and exhibit counter-gradient variation: provenances from northern latitudes flush earlier than populations from southern latitudes [18,19].

Clinal variations, either co- or counter-gradient, have usually been interpreted as consequences of divergent selection among populations by either abiotic or biotic selection pressures. For example, late-flushing trees will not suffer the detrimental effects of late frosts [20] or may avoid damage by defoliating insects [21,22]. However, few studies have considered the impacts of other evolutionary factors, such as gene flow in combination with the peculiar features of bud burst, in shaping the genetic variation of TBB. Indeed, because trees mate assortatively by flowering time [23,24], and because TBB is tightly linked to the timing of flowering, assortative mating is likely to shape the variation of TBB. Furthermore, under assortative mating, immigrant pollen will introduce genes likely to generate new allelic combinations for TBB, owing to the existence of environmental clines.

A number of theoretical studies have dissected the effects of assortative mating on the evolution of quantitative traits under polygenic inheritance, beginning with the early investigations by Fisher (1918) [25] and Wright (1921) [26]. All predicted that assortative mating will increase genetic variation as a result of the build up of genetic covariations among loci [25,27-29]. Others demonstrated the amplifying role of assortative mating on natural selection [24,30], as well as its contribution to allopatric speciation [31,32]. Finally, more recent studies aimed at predicting the effects of assortative mating on the genetic covariance of different traits [33-35]. No prior investigations, however, have considered the effects of assortative mating on a trait in multiple populations interconnected by extensive gene flow in the presence of environmental gradients. We tested whether interactions between gene flow and assortative mating under such circumstances could generate the distribution of genetic variation that is observed in common garden experiments, even in the absence of divergent selection. Our main hypothesis was that assortative mating, by filtering incoming alleles among interbreeding populations, will change the genetic composition and the genetic values of the phenological trait in recipient populations and hence generate population differentiation. We mainly focused on the maintenance of high within- and between-population genetic variation and on the build-up of genetic clines. There exists no available analytical theoretical prediction of genetic variation and differentiation taking into account assortative mating. We therefore used a simulation approach allowing us to monitor *in silico* the evolution of TBB under contrasting levels of assortative mating and environmental clines.

## Methods

### Components of population subdivision

Our main objective was to track components of genetic variation in phenology-related traits in a subdivided population that would mimic extant ecological settings. We were primarily interested in assessing the within- and between-population genetic variances (*V*_*W*_ and *V*_*B*_) as well as the differentiation among populations as measured by *Q*_*ST*_, which are standard genetic measurements used in quantitative genetics.

(1) $$Q_{\text{ST}}=\frac{V_{B}}{V_{B}+2V_{W}}$$

where *V*_*W*_ is the within-population genetic variance, and *V*_*B*_ is the between-population genetic variance. As suggested by recent QTL studies [9,36], we assumed that phenological traits were controlled by multiple QTLs with only additive effects. Previous theoretical studies have also shown that the genetic variances *V*_*B*_ and *V*_*W*_ of multilocus traits can be substantially inflated by allelic covariations among loci [37].

(2) $$V=\underset{i}{\sum }\sigma_{i}^{2}+\underset{i}{\sum }\underset{j\neq i}{\sum }\text{Co}v_{\text{ij}}$$

where $\sigma_{i}^{2}$ is the genic variance of locus *i* and *Cov*_*ij*_ is the covariance between allelic effects at locus *i* and *j*. *V * stands for *V*_*B*_ or *V*_*W*_ with appropriate $\sigma_{i}^{2}$ and *Cov*_*ij*_ expressed either at within- or between-population levels.

These covariations build up as a result of within- or between-gametic disequilibrium generated by different evolutionary forces and are scaled by the parameters *θ*_*W*_ and *θ*_*B*_.

(3) $$\theta =\frac{\overset{n}{\underset{i=1}{\sum }}\overset{n}{\underset{j\neq i}{\sum }}\text{Co}v_{\text{ij}}}{\overset{n}{\underset{i=1}{\sum }}\sigma_{i}^{2}}$$

Le Corre and Kremer (2003) [37] and Kremer and Le Corre (2011) [38] showed how the *θ* values contributed to the final differentiation of the trait together with the genetic differentiation that also arises at the QTLs controlling the trait ( $G_{ST_{q}}$).

(4) $$Q_{\text{ST}}=\frac{(1+\theta_{B})G_{ST_{q}}}{(\theta_{B}-\theta_{W})G_{ST_{q}}+1+\theta_{W}}$$

A major finding of previous theoretical work was that divergent selection generates important between-population disequilibria that becomes a major driver of population differentiation (*Q*_*ST*_) and has only a minor impact on differentiation at QTLs ( $G_{ST_{q}}$). In the absence of selection and under random mating, *θ*_*W*_ and _*θB*_ should be 0 and *Q*_*ST*_ equal to $G_{ST_{q}}$. We will explore in these simulations how assortative mating will shape the distribution of genetic variability by monitoring the different components of *Q*_*ST*_ (*e.g.V*_*W*_, *θ*_*W*_, *θ*_*B*_, *V*_*B*_, and $G_{ST_{q}}$) under different evolutionary scenarios.

### Models and simulations

We used the Metapop simulation engine to assess evolutionary changes along successive generations in a subdivided population. Essential steps of the evolutionary processes included in the software - mutation, gene flow, selection, demographic growth - have been described in earlier papers [37,39-41]. We will only address here the changes introduced to account for assortative mating and phenotypic clines of phenological traits.

#### Phenotypic subdivision of phenological traits

**Figure 1:**
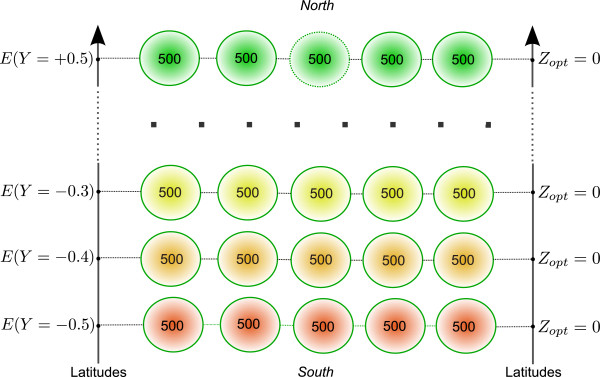
**Spatial settings of populations and environmental effects.** Fifty-five populations of 500 individuals each were spread homogeneously on a 5×11 grid along 11 latitudinal positions. *E*(*Y*) represents the environmental effect at a given latitude *Y * and is scaled by *k*_*E*_ (see equation 8). No selection was introduced: stabilizing selection was canceled with *ω*^2^ = 10^9^ and all populations shared a phenotypic optimum *Z*_*opt*_ = 0.

Populations are positioned on a two-dimensional grid (Figure 1) that mimics in a discrete way real situations showing continuous environmental variations. Each population is composed of *N* individuals. The overall phenotypic value *Z*_*ij*_^*′*^of individual *i* from population *j* is composed of three components: the additive part *G*_*ij*_ of the genes contributing to the trait, the environmental component *E*_*j*_ and a random local environmental deviation *∈*_*ij*_.

(5) $$Z_{\text{ij}}^{\prime }=G_{\text{ij}}+E_{j}+\in_{\text{ij}}$$

And the within-population phenotypic value is

(6) $$Z_{\text{ij}}=G_{\text{ij}}+\in_{\text{ij}}$$

*G*_*ij*_ is the genetic value resulting from the sum of additive effects of alleles present at *n* QTLs controlling the trait.

(7) $$G_{\text{ij}}=\overset{n}{\underset{l=1}{\sum }}{\left(\alpha_{1}+\alpha_{2}\right)}_{l}$$

*α* values are drawn at loci from the distribution $N(0,\sqrt{W_{l}*\sigma_{A_{0}}^{2}/2})$ where *W*_*l*_ is the level of contribution of the *l*th locus considered and $\sigma_{A_{0}}^{2}$ the initial variance of allelic effects based on estimated values of additive variance in experimental plantations. More details on the method are available in [38].

**Figure 2:**
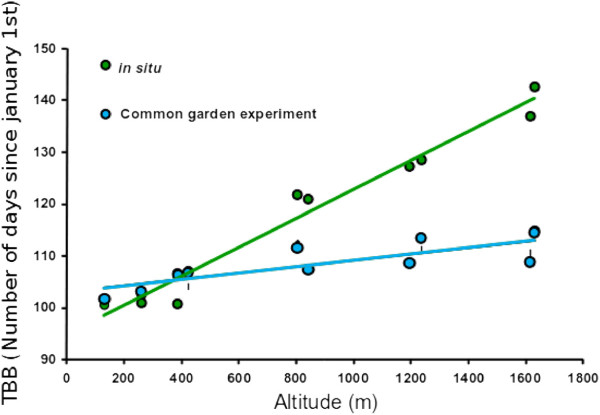
**An example of environmental and genetic clines for time of bud burst in oaks (data of Alberto et al., 2011 **[12]**).** The time of bud burst (TBB) was recorded in sessile oak (*Quercus petraea*) stands located along two valleys on the northern side of the Pyrénées mountains. *In situ* observations (green dots on the graph) showed that trees located at higher elevations flushed much later then trees located at lower altitudes, as a result of strong correlations between TBB and heat sum [4]. This pattern of variation, the phenotypic cline, is clearly linear. Open-pollinated seeds were collected in each stand and were experimentally raised in a common garden at low altitude, and TBB was monitored (blue points). The TBB was plotted as a function of the altitudes where the seeds were collected. A linear pattern of variation corresponds to a genetic cline. This example illustrates a co-gradient pattern of variation, because the slopes of the phenotypic and genetic clines share the same sign. Counter-gradient variation corresponds to cases where the two clines vary in opposite directions.

*E*_*j*_represents the influence of environmental conditions at the location of population *j*. *E*_*j*_ is of the same magnitude for all individuals of population *j* located at latitude *Y *. In our study case, *E* accounts for the effect of temperature on TBB demonstrated in forest trees [11]; indeed, flushing dates of broadleaves and conifers are tightly dependent on the heat sum [13] and exhibit continuous variation with latitude, resulting in environmental clines of *E* values. This is the rationale of assigning the same *E*_*j*_ value to all trees of population *j*. The linear variation of *E*_*j*_ along latitude, which corresponds to an environmental cline, results in the phenotypic cline as observed *in natura* (Figure 2). The steepness of the environmental cline is scaled by *k*_*E*_, a standardized measure of the between-environment variance relative to the within-population phenotypic variation. We considered different levels of steepness of the environmental cline by taking different values of *k*_*E*_.

(8) $$k_{E}=\frac{\sigma_{E}^{2}}{\left(\sigma_{G_{0}}^{2}+\sigma_{\in }^{2}\right)}$$

$\sigma_{G_{0}}^{2}$ being the total genetic variance observed within the initial population. Hence *k*_*E*_ is constant over the generations through the evolutionary process. Given that *E* follows a linear relationship with latitude, we can assign environmental values *E*_*j*_according to

(9) $$E_{j}=\sqrt{\frac{k_{E}\times (\sigma_{G_{0}}^{2}+\sigma_{\in }^{2})}{\sigma_{Y}^{2}}}\times Y_{j}$$

Finally, *∈*_*ij*_ is a random local environmental deviation following the distribution $N(0,\sigma_{\in })$.

#### Sequence of evolutionary processes in Metapop

Metapop implements evolutionary processes over successive generations in a subdivided population. Within each generation, processes are simulated along four steps within a main loop, depicted in Additional file 1: Figure S1. First, fitnesses of reproducing individuals are computed according to stabilizing and divergent selection. The level of stabilizing selection is scaled by the parameter *ω*^2^ from Turelli’s relation [42] while the strength of divergent selection is scaled by $\sigma_{Z_{\text{opt}}}^{2}$, where *Z*_*opt*_ of a given population is the phenotypic value for which trees have the highest fitness in that population. Second, from the populations growth settings and seed migration matrix, the number of individuals of each population contributing to the future generation is computed. Third, mates are chosen based on the constraints due to assortative mating scaled by the correlation between *Z*_*i*_^*′*^ and *Z*_*j*_^*′*^, the overall phenotypic values of individuals *i* and *j*.

(10) $$\rho =\frac{\text{cov}(Z_{i}^{\prime },Z_{j}^{\prime })}{\sigma_{Z_{i}^{\prime }}\times \sigma_{Z_{j}^{\prime }}}$$

Following 10, the differences in phenotypic values of two mating parents are drawn from the distribution $N(0,\sigma_{\delta }$) with

(11) $$\sigma_{\delta }=\sqrt{\frac{\sigma_{Z_{i}^{\prime }}^{2}}{\rho^{2}}-\sigma_{Z_{i}^{\prime }}^{2}}$$

Fertilization occurs by drawing male and female gametes conditionally to *ρ*, fitness of the parents and seeds migration matrix. A proportion of male gametes, based on the pollen migration matrix, is drawn from other populations to account for pollen flow. Finally, mutation is also considered.

#### Monitoring of gene flow

We now consider how the interaction between gene flow and assortative mating may modify the genetic values in natural populations. Because assortative mating will filter immigrant alleles so that they can mate with trees of recipient populations, we compare the genetic values of immigrant alleles to local alleles to explore whether gene flow will modify the mean genetic value of populations.

In each generation, matings take place between trees of the same population, but a fraction *m*_*p*_ of matings involves pollen from other populations. We can subdivide the genetic value of the offspring into two components:

(12) $$G_{t+1}=(1-m_{p})(\frac{1}{2}G_{t}^{\text{♀}}+\frac{1}{2}G_{t}^{\text{♂}})+m_{p}(\frac{1}{2}G_{t}^{\text{♀}}+\frac{1}{2}G_{t}^{*})$$

where *G*_*t*_^♀^ and *G*_*t*_^♂^stand respectively for the mean genetic values of the female and male parents, and $G_{t}^{*}$ stands for the mean genetic value of the male parents providing immigrant alleles at generation *t*. $\left(1-m_{p})(\frac{1}{2}G_{t}^{\text{♀}}+\frac{1}{2}G_{t}^{\text{♂}}\right)$ represents the component of the genetic value due to intra-population matings and $m_{p}(\frac{1}{2}G_{t}^{\text{♀}}+\frac{1}{2}G_{t}^{*})$ the component of the genetic value due to inter-population matings involving external incoming alleles. Each generation, $G_{t}^{\text{♀}}=G_{t}^{\text{♂}}=G_{t}$. When assortative mating occurs within populations, mating parents share similar phenotypic values, and because they belong to the same population, they also share the same environmental values. However, because male parents from the outside populations should share the same phenotypic value as the female parent, their genetic values are likely to be different from those of the female parents owing to the environmental gradient. Within a population, the mean phenotypic value of the male parents corresponding to the immigrant alleles is equal to

(13) $${Z^{\prime }}_{t}^{*}=G_{t}^{*}+E^{*}$$

and the mean phenotypic value of the female parents is equal to

(14) $$Z_{t}^{\prime }=G_{t}+E$$

Because the phenotypic values of both parents should be similar owing to assortative mating, the mean genetic value of the male parents is

(15) $$G_{t}^{*}≃G_{t}+E-E^{*}$$

As a result, each generation the genetic value of the population is expected to shift by about *Δ*=*G*_*t* + 1_−*G*_*t*_, which can be expressed in

(16) $$\Delta ≃\frac{1}{2}m_{p}(E-E^{*})$$

More generally, matings that occur within populations can be subdivided in two different kinds: (1) matings between individuals sharing similar genetic values, which would correspond to positive assortative mating and (2) matings between individuals likely to have different genetic values resulting from gene flow. In the extreme case, these matings may result from negative assortative mating. The shift of the genetic value is therefore driven by the level of effective gene flow *m*_*p*_ and the difference in environmental values between the recipient and donors populations. Consequently, we monitored the effective pollen flow during the simulations by tracking its spatial origin.

#### Simulations settings

We simulated the evolution of 55 populations of 500 individuals each spread homogeneously on a 5 × 11 grid depicted in Figure 1. We did not consider overlapping generations and the number of individuals per population was kept constant over successive generations. A fictive gradient of latitudes was set from latitude −0.5 to latitude + 0.5 in steps of 0.1. Three levels of environmental clines were considered along the latitudinal gradient: *k*_*E*_ = 1, *k*_*E*_ = 2 and *k*_*E*_ = 3.

Recent observations in oak populations suggested that assortative mating for TBB is substantial [6]. Indeed, the flowering time in oak may extend over several weeks within a population, but the receptive period of female flowers lasts only a few days at the individual level. We consequently investigated two strengths of assortative mating, encompassing the suspected range of variation, using *ρ* = 0.3 and *ρ* = 0.8 to model moderate and strong assortative mating, respectively. Random mating was considered as well with *ρ* = 0.

We used Wright’s island migration model to generate gene flow among populations located on the grid system, and considered two levels of gene flow: *Nm* = 5.1 and *Nm* = 10.2. These values fit the range of variation of *F*_*ST*_ values (2.4% to 4.7%) observed in natural oak populations [7]. Pollen and seed migration rates (*m*_*p*_ and *m*_*s*_) were then inferred from *Nm* values and introduced in the simulations, assuming further that *m*_*p*_ = 100∗*m*_*s*_ (Table 1). In addition to the island model, we also designed gene flow via the stepping stone model using pollen and seed migration rates corresponding to *Nm* = 5.1.

**Table 1:** Initial simulation settings

	
heritability *h*^2^	0.83
selfing rate *s*	0.02
nb. of populations *d*	55
nb. of ind. per pop. *N*_*ind*_	500
pollen migration rates *m*_*p*_	0.02, 0.04
seed migration rates *m*_*s*_	0.0002 , 0.0004
nb. of QTL *n*	10
mutation rate *μ*	10−^5^
nb. of latitude levels *Y*	11
interval of latitudes *Y*	$\left[-0.5,+\;0.5\right]$
steepness of environmental cline *E* scaled by *k*_*E*_	1, 2, 3
variance of *Z*_*opt*_ across latitudinal levels $\sigma_{Z_{\text{opt}}}^{2}$	0, 1
intensity of stabilizing selection *ω*^2^	10^9^, 5
assortative mating intensity *ρ*	0; 0.3; 0.8

Assuming that the starting populations were in mutation-migration-drift equilibrium, initial allelic frequencies in different populations were drawn from a Dirichlet distribution [38]. We assumed that phenological traits were controlled by 10 QTLs. Additive values of alleles were chosen at random from a Gaussian distribution whose initial variance was adjusted to fit the heritability values observed in extant progeny plantations, 0.83 from [43]. Mutations at each QTL occurred across generations at a rate of *μ* = 10−^5^ per generation. The local environmental deviation was drawn at random from the distribution N(0,1).

We considered eight different evolutionary scenarios by combining unique slopes of environmental clines, levels of assortative mating, migration models, and levels of gene flow (Table 2). Because our investigations were focused on the impact of gene flow and assortative mating on the evolution of TBB, we purposely excluded selection in the simulations. We consequently canceled stabilizing selection within all populations by setting all *ω*^2^ values to 10^9^, and we set all *Z*_*opt*_ values to 0. However, as a control, we added one scenario including selection (*ω*^2^ = 5 and $\sigma_{Z_{\text{opt}}}^{2}=1$), corresponding to strong stabilizing selection and moderate divergent selection. This scenario did not consider assortative mating and was designed to compare the steepness of the genetic clines observed in the eight studied cases with a selective scenario. For each evolutionary scenario based on combinations of these settings (Table 2), we performed 50 independent replicated simulations over 1000 generations.

**Table 2:** Evolutionary scenarios

	***ρ = *0**	***ρ = *0.3**	***ρ = *0.8**
*k*_*E*_ = 1	×^∗^	×	×
*k*_*E*_ = 2	×^∗^	×	×,×^*s*^,×^*m*^
*k*_*E*_ = 3			×

^*^identical scenarios; because under random mating, phenotypic values of individuals have no impact on our simulation outcomes, variations in the environmental component have no influence when *ρ* = 0. ^×*s*^ and ×^*m*^stand respectively for scenarios simulated under the stepping-stone migration model and with a higher migration rate (*Nm* = 10.2) under the island migration model.

## Results

### Within population genetic variance

**Figure 3:**
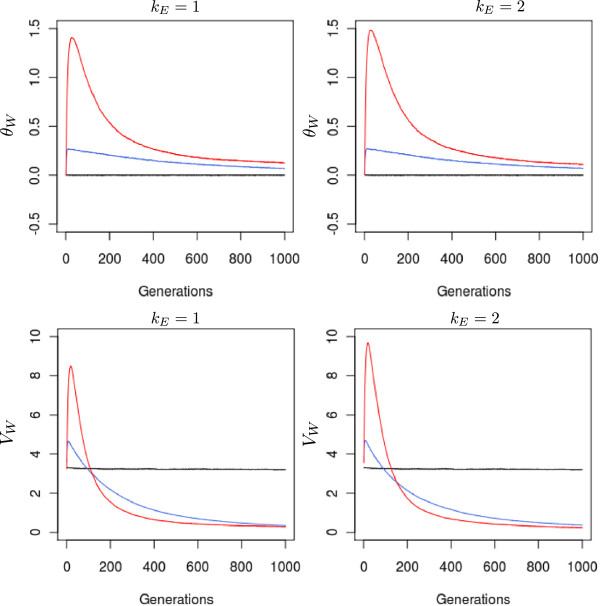
**Variations in within-population allelic covariation (***θ***_*W*_) and genetic variance (*V*_*W*_) under different evolutionary scenarios. ***θ*_*W*_ and *V*_*W*_ were monitored under three different strengths of assortative mating and two levels of environmental cline. All simulations were conducted under the island migration model with moderate gene flow (*Nm* = 5.1). The red line indicates strong assortative mating (*ρ* = 0.8), the blue line moderate assortative mating (*ρ* = 0.3), and the black line random mating (*ρ* = 0). Each line represents the mean of 50 independent replicates for each evolutionary scenario.

Assortative mating substantially increased allelic covariances during the first generations (Figure 3). After reaching maximum values, covariances decreased very rapidly and evolved to asymptotic levels. These patterns were more pronounced when assortative mating was strong and were only slightly modified by the magnitude of the environmental cline. Under strong assortative mating, covariances accounted for more than 1.5 of the genic variances relative to the total genetic variance, while under moderate assortative mating, the maximum value was only 0.28. Under steeper clines, the maximum values of *θ*_*W*_ were slightly higher, 1.5 *vs* 1.4, and its change over generations was slightly delayed. Overall *θ*_*W*_ values were always larger under assortative mating than under random mating.

The variations in *θ*_*W*_ had striking consequences on the genetic variances (equation 2). Indeed, under assortative mating, genetic variances increased rapidly during the early generations, then they very rapidly dropped below even the level of genetic variance reached under random mating. As for covariances, there was a strong effect of the level of assortative mating and only a minor effect of the environmental cline. The decrease in genetic variance due to assortative mating could be dramatic after 400 generations. Furthermore, the final heritability for the trait was divided by a factor 2.5 at generation 500. As expected without selection in large populations, genetic variance was maintained under random mating and extensive gene flow.

### Between population genetic variance

**Figure 4:**
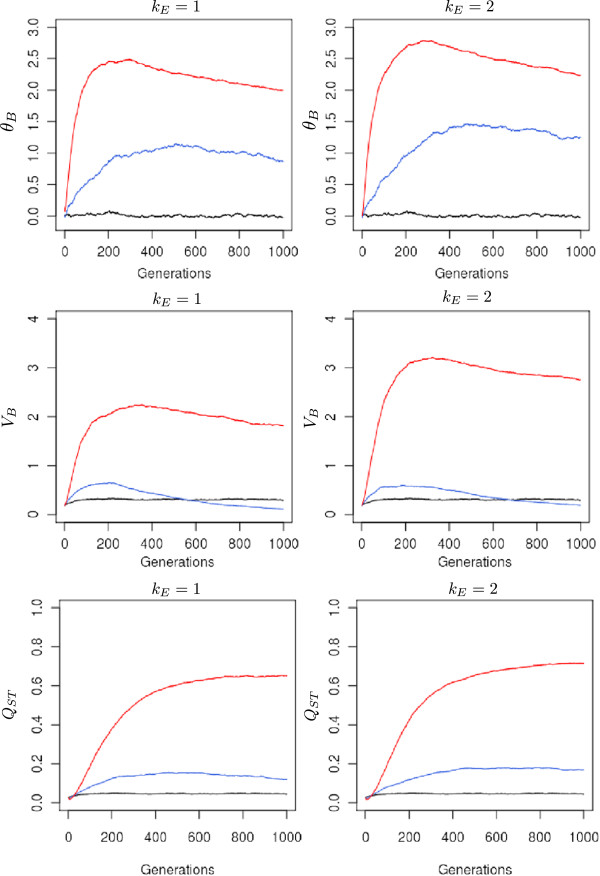
**Variations in between-population allelic covariation (*θ*_*B*_), between-population variation (*V*_*B*_), and differentiation of TBB (*Q*_*ST*_).** These measurements were monitored under three different strengths of assortative mating and two levels of environmental cline. All simulations were conducted under the island migration model with moderate gene flow (*Nm* = 5.1). The red line indicates strong assortative mating (*ρ* = 0.8), the blue line moderate assortative mating (*ρ* = 0.3), and the black line random mating (*ρ* = 0). Each line represents the mean of 50 independent replicates for each evolutionary scenario.

Assortative mating had a strong effect on allelic covariances at the between-population level; _*θB*_increased during the early generations and was maintained at higher values through the 1000 generations, in contrast to *θ*_*W*_ values. There was a stronger impact when environmental clines were steeper. For example, under strong assortative mating, the maximum value of *θ*_*B*_ was 2.7 when *k*_*E*_ = 2*vs* 2.5 when *k*_*E*_ = 1. The initial phase of increase lasted longer under moderate assortative mating than under strong assortative mating: 500 generations *vs* 230 generations when *k*_*E*_ =  1 (Figure 4).

Between-population variances of allelic frequencies at selected loci increased steadily over generations. They increased more rapidly under strong assortative mating, while no substantial differences were observed between random mating and moderate assortative mating. By generation 1000, differentiation at selected loci had reached 0.16, which could be compared with differentiation under random mating (0.03), which was very close to differentiation at neutral markers (0.024) (data not shown). Overall, between-population genetic variances exhibited strong differences between moderate and strong assortative mating and also between low and strong environmental clines (Figure 4).

### Trait differentiation and genetic clines

Because assortative mating had strong consequences on within- and between-population genetic variances, it ultimately contributed to population differentiation of the trait. There were striking differences in the levels of differentiation observed under random and assortative mating. *Q*_*ST*_ values steadily increased under assortative mating and reached up to 0.7 when *k*_*E*_ = 2. There was only a slight effect of the steepness of the environmental cline on the level of differentiation: *Q*_*ST*_ = 0.7 when *k*_*E*_ = 2*vs* 0.62 when *k*_*E*_ = 1.

**Figure 5:**
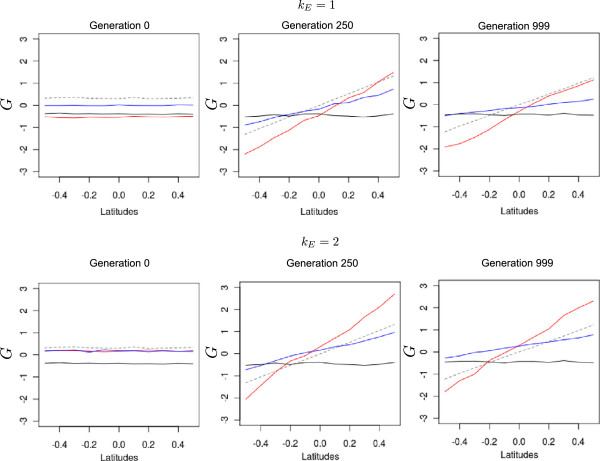
**Variations in mean population genetic values at different latitudes and at different generations.** The value for each latitude is the average of the five mean genetic values for the populations concerned. Latitudinal means were computed and reported for two levels of environmental cline and three different strengths of assortative mating. All simulations were conducted under the island migration model with moderate gene flow (*Nm* = 5.1). The red line indicates strong assortative mating (*ρ*=0.8), the blue line moderate assortative mating (*ρ* = 0.3), and the black line random mating (*ρ* = 0). The dashed line depicts the mean genetic value obtained under divergent selection modeled with *ω*^2^ = 5 and $\sigma_{Z_{\text{opt}}}^{2}=5$ and without assortative mating. Each line represents the mean of 50 independent replicates for each evolutionary scenario.

**Figure 6:**
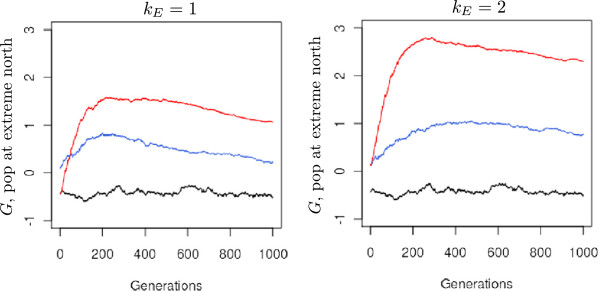
**Evolution of the mean genetic value of a population located at the extreme north of the landscape.** The mean genetic value of a population located at latitude + 0.5 (dotted circle in Figure 1) was monitored under two different levels of environmental cline and three different strengths of assortative mating. All simulations were conducted under the island migration model with moderate gene flow (*Nm*=5.1). The red line indicates strong assortative mating (*ρ*=0.8), the blue line moderate assortative mating (*ρ*=0.3), and the black line random mating (*ρ* = 0). Each line represents the mean of 50 independent replicates for each evolutionary scenario.

This effect was due to the trade-off between variations in *V*_*B*_ and *V*_*W*_ in equation 1. The steepness of the environmental cline increased *V*_*W*_ (Figure 3) and had a decreasing effect on *Q*_*ST*_, but at the same time, it also increased *V*_*B*_, increasing *Q*_*ST*_ (Figure 4). As a result, *Q*_*ST*_ showed similar values at both levels of environmental cline. These results suggested that assortative mating differentiated populations and shifted their mean genetic values. We consequently examined the spatial distribution of mean genetic values across the landscape; indeed, a cline of genetic values built up during the early generations following a south-north gradient (Figure 5). The steepness of the genetic cline was stronger under assortative mating and under steep environmental clines resulting in a co-gradient variation with the environmental cline. The temporal dynamics of the cline could be illustrated by the changes in the genetic value of the population located at the extreme northern latitude (Figure 6). This value reached a peak between generation 200 and 400, depending on the steepness of the environmental cline and the level of assortative mating. No genetic cline developed under random mating.

**Figure 7:**
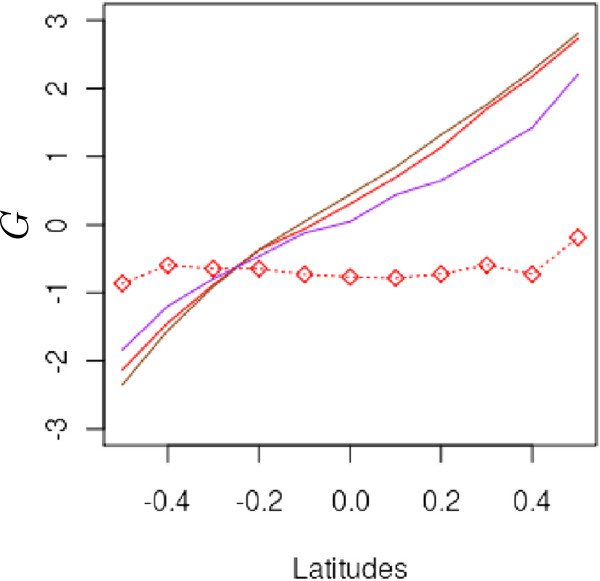
**Variations in mean population genetic values at different latitudes under multiple scenarios.** The value for each latitude is the average of the five mean genetic values for the populations concerned at generation 300. All scenarios (except the selection scenario, dashed line) were conducted under strong assortative mating (*ρ* = 0.8). Red line: steep environmental cline (*k*_*E*_ = 2), island migration model, moderate gene flow (*Nm* = 5.1). Purple line: very steep environmental cline (*k*_*E*_ = 3), island migration model, moderate gene flow (*Nm* = 5.1). Brown line: steep environmental cline (*k*_*E*_ = 2), island migration model, extensive gene flow (*Nm*=10.2). Red line with open circles: steep environmental cline (*k*_*E*_ = 2), stepping stone migration model, high gene flow (*Nm*=5.1). Dashed line: random mating, divergent selection ( $\sigma_{Z_{\text{opt}}}^{2}=1$), strong stabilizing selection (*ω*^2^ = 5), without assortative mating. Each line represents the mean of 50 independent replicates for each evolutionary scenario.

**Figure 8:**
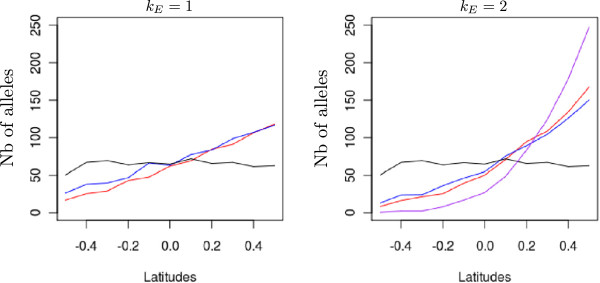
**Amount of immigrant alleles received by a northern population.** Absolute number of immigrant alleles into a population located at the extreme northern latitude (+0.5 dotted circle in Figure 1). Numbers on the y-axis are cumulative counts of alleles from generation 16 to 20. Counts of alleles were monitored at three strengths of assortative mating and three levels of the environmental cline. The red line indicates strong assortative mating (*ρ* = 0.8), the blue line moderate assortative mating (*ρ* = 0.3), the black line random mating (*ρ* = 0), and the purple line strong assortative mating under an extreme environmental cline (*ρ* = 0.8, *k*_*E*_ = 3). Lines are mean values of 50 replicates for each evolutionary scenario.

**Figure 9:**
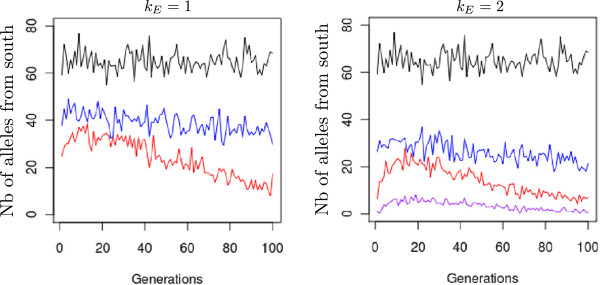
**Amount of southern immigrant alleles received by a northern population over generations.** Absolute number of immigrant alleles into a population located at the extreme northern latitude (+0.5 dotted circle in Figure 1) and coming from southern latitudes (-0.5 to -0.1). Only gene flow between populations is represented here. Numbers on the y-axis are counts of alleles at a given generation (x-axis). Counts of alleles were monitored at three strengths of assortative mating and three levels of environmental cline. All simulations were conducted under the island migration model with moderate gene flow (*Nm* = 5.1). The red lines indicate strong assortative mating (*ρ* = 0.8), the blue line indicates moderate assortative mating (*ρ* = 0.3), the black line random mating (*ρ*  = 0), and the purple line strong assortative mating under an extreme environmental cline (*ρ* = 0.8, *k*_*E*_ = 3). Lines are mean values of 50 replicates for each evolutionary scenario.

**Figure 10:**
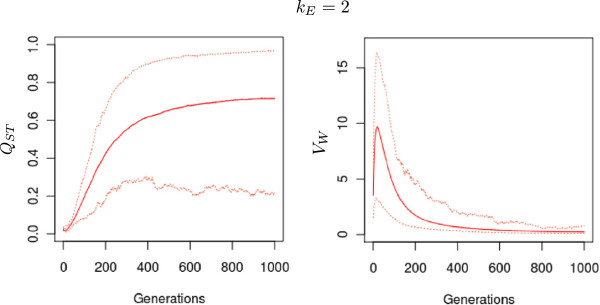
**Stochastic variations in *Q***_***ST ***_**and *VW*among different simulations within a given scenario.** Upper and lower bounds of the 50 simulations conducted per scenario. *ρ* was set to 0.8 in all cases. *k*_*E*_ is the scaling factor of the environmental cline. Plain lines indicate mean values of the 50 simulations for each scenario and dotted lines represent the two simulations that gave the extreme results.

We also explored the clinal patterns resulting from a more extreme environmental cline, a higher migration rate, and the stepping-stone migration model (Figure 7). Surprisingly the resulting genetic cline was less pronounced under *k*_*E*_ = 3 than under *k*_*E*_ = 2. When *k*_*E*_ = 3, the environmental variance among populations was 3-fold larger than the within-phenotypic variance (equation 8). Consequently, phenological matches between trees from different populations were limited, thus increasing the filtering of incoming genes to proximal populations (Figures 8 and 9). Similarly, when the pollen dispersal distance was *a priori* reduced to the most proximal populations, as in the stepping-stone migration model, a very shallow genetic cline built up (Figure 7). In this latter case, when *Nm* = 5.1, *ρ* = 0.8, and *k*_*E*_ = 2, only populations at extreme latitudes became genetically differentiated. Despite this very shallow cline, *Q*_*ST*_ approached 0.45 at generation 1000 under the stepping-stone migration model; under the same simulations parameters, *Q*_*ST*_ values reached 0.7 under the island migration model. Finally, when pollen migration rates increased (*Nm* = 10.2*vsNm* = 5.1), no significant change was observed in the slopes of the clines. However, additional investigations indicated that lower migration rates decreased the slopes of the genetic clines and induced higher _*QST*_values, owing to an important drift effect [37] (Additional file 2: Figure S2 and Additional file 3: Figure S3). Overall large stochastic variations were associated with the genetic parameters that were monitored during the evolutionary scenarios (data not shown). We illustrate these variations only for *Q*_*ST*_ and *V*_*W*_ (Figure 10). The trend among generations, *i.e.*, the form of the curve, was the same among the replicates.

### Pollen filtering by assortative mating

We monitored the incoming pollen composition in a population located at the extreme northern latitude. By doing so, we expected to predict the shift in genetic values that contributed to the development of the genetic cline under the island migration model (equation 16). Figure 8 clearly shows that assortative mating filtered incoming alleles by geographic origin. Very rapidly, there was a preferential screening of incoming alleles from neighboring populations in the case of assortative mating, and the trend was more pronounced when the environmental cline grew steeper. The discrepancy between distant and proximal alleles was more pronounced with strong assortative mating. Furthermore, the level of filtering changed over generations. More alleles arrived from distant populations during the first 40 generations, especially when strong assortative mating was occurring (Figure 9). These distant alleles would shift the genetic values of populations as predicted by *Δ*.

## Discussion

Our simulations demonstrated that genetic clines could be established in the absence of divergent selection. We showed that the combination of assortative mating and pre-existing environmental clines resulted in population genetic differentiation along the environmental cline. We also confirmed that assortative mating increased the within-population genetic variances in the early stages of the evolutionary scenarios. However, assortative mating was also responsible for the severe decline in genetic variation in later generations.

These patterns resulted in a positive covariance between genetic and environmental population values and corresponded to what has been called co-gradient variation [14,15]. We discuss here how such covariations may build up under assortative mating in the case of phenological traits in trees. Given the pre-existence of environmental clines, genetic clines are generated by the combined effects of assortative mating and gene flow. In particular, we examine how the interplay between assortative mating and gene flow will actually produce the genetic cline we observed. According to equation 15, the larger the physical distance between the mates associated by gene flow, the more different their genetic values. As a consequence, a larger shift in the mean genetic value should be expected at extreme latitudes in our grid settings (Figure 1). In what follows, we illustrate this trend by providing values for the shift *Δ* obtained at the extreme northern latitude under the strongest assortative mating intensity and across the steepest environmental cline.

We can subdivide the evolutionary process into three main phases, illustrated in Figures 4, 5, 6, 7 and 8. 

(1) In the very early generations (05), the mean genetic value is 0 for all populations, there is no within-population allelic covariance, and alleles are randomly spread over the landscape. During this period, assortative mating will generate phenotypes with extreme genetic values in each population. Hence the genetic variance within populations increases as predicted by previous analytical models [24,34] and numerical simulations [28,32,44]. Gene flow during the early generations preferentially imports alleles from neighboring populations (Figure 8), owing to the fact that populations at this stage are genetically undifferentiated over the whole grid and parents exhibiting similar phenotypes are more likely to be in neighboring populations. As a result, the shift *Δ*remains limited: 0.0798 at the allelic level for northern populations.

(2) From generation 5 to about 30, because the increase in within-population genetic variance has now produced phenotypes with more extreme values, gene flow tends now to import alleles from more distant populations (Figure 9). The fraction of imported alleles enriches the population gene pool and further facilitates an increase in genetic covariances *θ*_*W*_. The genetic variance between populations continues to increase steadily. During the second phase, the *Δ*value tends to be larger (0.14) as a result of more divergent alleles imported by distant gene flow. A similar effect that symmetrically decreases the *Δ*value of incoming gene flow within southern populations is expected to take place at the same time. As a result, the mean genetic values of the population shift strongly, leading to the progressive formation of the genetic cline.

(3) After generation 30, most of the alleles have been spatially redistributed by gene flow constrained by assortative mating at the landscape level. Allelic covariations within populations have been exhausted and the genetic variance has now reached its maximum. Assortative mating within populations tends now to become a selective factor favoring phenotypes following the shift of the mean genetic values. Furthermore, gene flow again becomes strongly restricted to neighboring populations that share fewer divergent alleles than distant populations. Restricted gene flow therefore reinforces the decrease in the genetic variance. Overall, phase 3 is characterized by a continuous decrease in genetic variance and the reaching of an asymptotic mean genetic value in populations; the genetic cline is establishing. We further advocate that restricted gene flow, together with within-population assortative mating, now constrains effective population sizes, accelerating the decrease in genetic variance due to drift. A similar decrease was observed by Devaux and Lande [32] in a single population, despite a high mutation rate. Jorjani et al. also noticed a decreasing effect of negative assortative mating on the evolution of the genetic variance within a single population [44].

These three phases were observed for all of the simulation settings we used. The lengths of the two first phases extended over longer periods, populations differentiated more rapidly, and genetic clines were shaped faster under strong assortative mating. By dissecting the evolutionary process, we showed that the screening of immigrant alleles due to assortative mating triggers shifts in the genetic values of populations (Figures 6 and 9). Indeed, when assortative mating allows for long-distance filtered pollen flow, the shifts in the genetic values of recipient populations are strongly enhanced. Because moderate assortative mating generates less extreme genotypes over generations, distant gene flow is promoted less and the mean expected shift in the mean genetic values of populations remains limited. Consequently, under moderate assortative mating, the final steepness of genetic clines is less dependent on the steepness of environmental clines (Figures 5 and 6).

Increasing the slope of the environmental cline generated more genetic variance and higher genetic differentiation as well. According to equation 15, each generation steeper environmental clines increase the expected divergence between mates from distinct populations. However, the divergence is constrained by the necessary overlap of parental flowering times. If long distance pollen flow is restricted by large phenological differences among populations, then assortative mating will favor matings between proximal populations, and the shift in genetic values will be limited. In our simulations, the latter case occurred when we explored very large *k*_*E*_ values (*k*_*E*_ = 3).

A similar outcome was observed under the stepping-stone migration model. In this case, populations do not differentiate except at the northern and southern edges of the landscape (Figure 7). This result is only partly explained by the absence of distant gene flow. Indeed, according to the expression of *Δ*and considering the features of the stepping-stone migration model, limited *Δ* values are expected owing to pollen flow from adjacent latitudes. However, incoming alleles from neighboring northern populations balance with incoming alleles from neighboring southern populations. As a consequence, the shift induced within populations by southern gene flow is systematically canceled by the one caused by northern flow, resulting in a null contribution to the *Δ* values. Finally, because under the stepping-stone migration model, incoming gene flow is latitudinally unbalanced at the northern and southern margins of the grid, the genetic values of populations can be shifted by assortative mating at these latitudes. These results suggest that the spatial configuration of the populations in combination with the migration model may also contribute to the building of the genetic cline. Any combination that increases an asymmetry in gene flow between northern and southern populations will enhance the genetic cline, while symmetry will tend to even out the effects of northern and southern gene flow.

To summarize, the construction of a genetic cline as a result of the combined effects of gene flow and assortative mating can only be met under certain circumstances when there is a balance between the intra-population and between-population phenotypic variance (*k*_*E*_ varying between 1 and 3), when long distance pollen flow is possible, and when the patterns of incoming pollen flow at population level are unbalanced regarding the environmental cline. Interestingly these criteria are met under realistic situations. Taking oaks as an example, flushing dates may vary over 5 weeks from southwestern to central France [4], while the same range of variation may be observed between early and late flushing trees in a given forest stand. Viable pollen has also been shown to be dispersed over such distances [45].

## Conclusions

Our simulations showed that interaction between assortative mating and gene flow across environmental clines may shape the genetic variability of phenologically-related traits and induce cogradient variation without any divergent selection. We also demonstrated that the extent of genetic variability resulting from assortative mating was related to the patterns of incoming pollen flow at the population level. Because phenotypic clines have been very widely reported in forest trees [2,11,18], we suspect that assortative mating and gene flow could actually be responsible for the co-gradient variation observed in some species in common garden experiments [12,17]. However, most tree species actually exhibit counter-gradient variation [46,47], suggesting that other evolutionary forces, such as divergent selection, actually counteract the combined effects of assortative mating and gene flow. In a subsequent paper, we will explore how selection interacts with assortative mating and gene flow to generate counter-gradient variation. Finally, our simulations also indicated that very large levels of genetic variation should also be expected within populations, generated by genetic covariances in allelic effects due to assortative mating as predicted by other theories or simulations [24,25,32]. Experimental data from progeny tests of forest trees indeed show that heritability values of phenologically-related traits can exceed 0.5, much larger than other phenotypic traits generally assessed in experimental plantations [43]. Furthermore, our simulations predict that the steep increase in genetic variation will be temporary and will be followed by a rapid decrease. Once all covariation has been exhausted, assortative mating will act as a selective force by constraining the synchronicity of male and female flowering periods. Given the large genetic variation still existing in extant forest stands, we suspect that the time of decrease has not yet been reached in natural populations, owing to the long generation times of trees. Finally, our simulations should be prolonged under more realistic ecological settings, including different patterns of gene flow and selection on multiple traits. Both authors read and approved the final manuscript.

## Competing interests

The authors declare that they have no competing interests.

## Author’s contributions

JPS and AK designed the study and identified the different evolutionary scenario to be tested. JPS adapted the Metapop simulation engine by introducing assortative mating and environmental clines. JPS and AK wrote the paper. Both authors read and approved the final manuscript.

## Supplementary Material

Additional file 1 Figure S1. Summary of the evolutionary processes within a generation. Fitness values and sizes of populations are first computed according to selection settings, demographic settings, and the seed migration matrix. Reproduction takes place between mates paired according to fitness, seed migration settings, and pollen migration settings. Assortative mating may bear additional iterations for the choice of male and female parents because mates must share close phenotypic values. Mutations may occur. Click here for file

Additional file 2 Figure S2. Variations in mean population genetic values at different latitudes under a range of migration rates. The value for each latitude is the average of the five mean genetic values for the populations concerned at generation 300. All scenarios were conducted under strong assortative mating (*ρ* = 0.8), island migration model and steep environmental cline (*k*_*E*_ = 2). Brown line: *Nm* = 10.2, red line: *Nm* = 5.1, green line: *Nm* = 1, green dashed line: *Nm* = 0.5 and green dotted line: *Nm* = 0.1. Each line represents the mean of 50 independent replicates for each evolutionary scenario. Click here for file

Additional file 3 Figure S3. _*QST*_values after 1000 generations under a range of migration rates. All simulations were conducted under under strong assortative mating (*ρ* = 0.8), island migration model and steep environmental cline (_*kE*_ = 2). Brown line: *Nm* = 10.2, red line: *Nm* = 5.1, green line: *Nm* = 1, green dashed line: *Nm* = 0.5 and green dotted line: *Nm* = 0.1. Each line represents the mean of 50 independent replicates for each evolutionary scenario. Click here for file
