# Supplementary material for: Assortative mating and gene flow generate clinal phenological variation in trees
Source: BMC Evol Biol. 2012 Jun 8;12:79. doi: 10.1186/1471-2148-12-79 (PMC3541993; doi:10.1186/1471-2148-12-79)
Supplement: Additional file 1 — Figure S1. Summary of the evolutionary processes within a generation. Fitness values and sizes of populations are first computed according to selection settings, demographic settings, and the seed migration matrix. Reproduction takes place between mates paired according to fitness, seed migration settings, and pollen migration settings. Assortative mating may bear additional iterations for the choice of male and female parents because mates must share close phenotypic values. Mutations may occur. [file 1471-2148-12-79-S1.pdf]

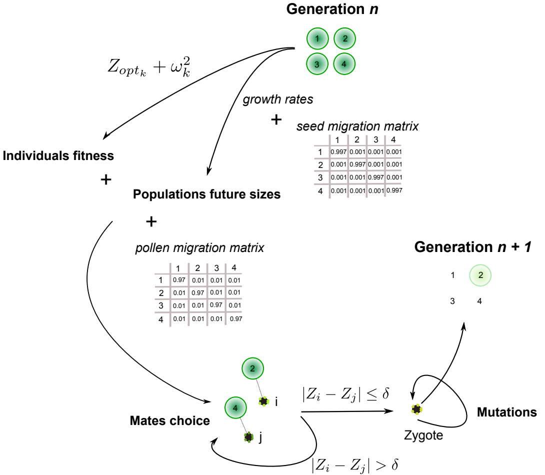

Generation *n*
1 2 3 4
$Z_{opt_k} + \omega_k^2$
growth rates
+
seed migration matrix
1 2 3 4
1 0.997 0.001 0.001 0.001
2 0.001 0.997 0.001 0.001
3 0.001 0.001 0.997 0.001
4 0.001 0.001 0.001 0.997
Individuals fitness
+
Populations future sizes
+
pollen migration matrix
1 2 3 4
1 0.97 0.01 0.01 0.01
2 0.01 0.97 0.01 0.01
3 0.01 0.01 0.97 0.01
4 0.01 0.01 0.01 0.97
Generation *n + 1*
1 2
3 4
2
4
i
j
Mates choice
$|Z_i - Z_j| \leq \delta$
$|Z_i - Z_j| > \delta$
Zygote
Mutations
