# Supplementary figures and images for: Assortative mating and gene flow generate clinal phenological variation in trees
Source: BMC Evol Biol. 2012 Jun 8;12:79. doi: 10.1186/1471-2148-12-79 (PMC3541993; doi:10.1186/1471-2148-12-79)

$$k_E = 2$$

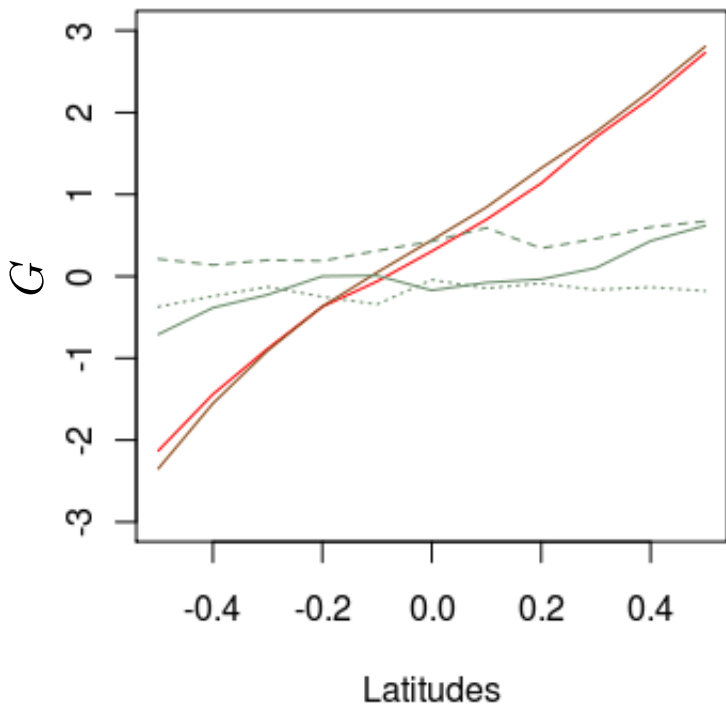

Supplement: Additional file 2 — Figure S2. Variations in mean population genetic values at different latitudes under a range of migration rates. The value for each latitude is the average of the five mean genetic values for the populations concerned at generation 300. All scenarios were conducted under strong assortative mating (ρ = 0.8), island migration model and steep environmental cline (kE = 2). Brown line: Nm = 10.2, red line: Nm = 5.1, green line: Nm = 1, green dashed line: Nm = 0.5 and green dotted line: Nm = 0.1. Each line represents the mean of 50 independent replicates for each evolutionary scenario. [file 1471-2148-12-79-S2.pdf]

$$k_E = 2$$

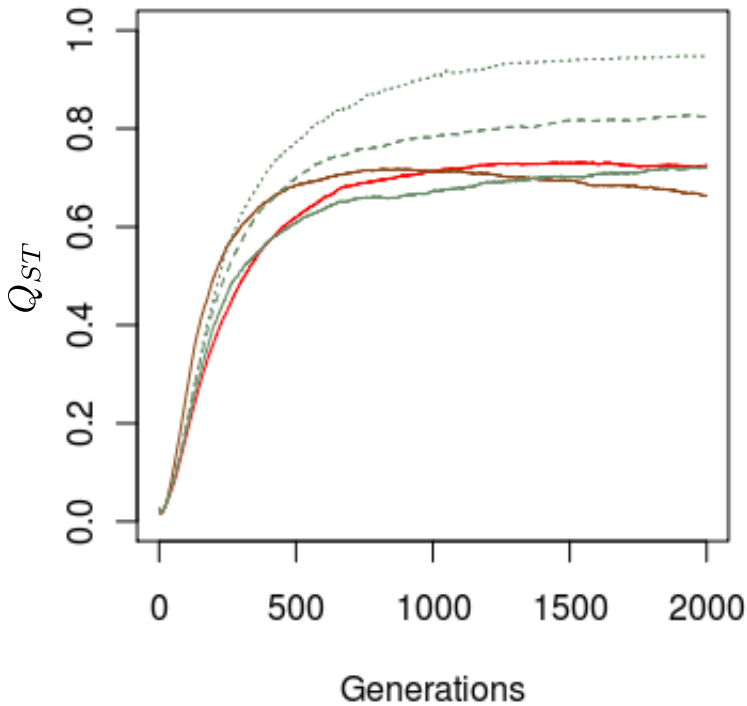

Supplement: Additional file 3 — Figure S3. QSTvalues after 1000 generations under a range of migration rates. All simulations were conducted under under strong assortative mating (ρ = 0.8), island migration model and steep environmental cline (kE = 2). Brown line: Nm = 10.2, red line: Nm = 5.1, green line: Nm = 1, green dashed line: Nm = 0.5 and green dotted line: Nm = 0.1. Each line represents the mean of 50 independent replicates for each evolutionary scenario. [file 1471-2148-12-79-S3.pdf]
